# The RACK1 Signaling Scaffold Protein Selectively Interacts with *Yersinia pseudotuberculosis* Virulence Function

**DOI:** 10.1371/journal.pone.0016784

**Published:** 2011-02-10

**Authors:** Sara E. Thorslund, Tomas Edgren, Jonas Pettersson, Roland Nordfelth, Mikael E. Sellin, Ekaterina Ivanova, Matthew S. Francis, Elin L. Isaksson, Hans Wolf-Watz, Maria Fällman

**Affiliations:** 1 Department of Molecular Biology, Umeå University, Umeå, Sweden; 2 Umeå Centre for Microbial Research (UCMR), Umeå University, Umeå, Sweden; Institut de Pharmacologie et de Biologie Structurale, France

## Abstract

Many Gram-negative bacteria use type III secretion systems to translocate effector proteins into host cells. These effectors interfere with cellular functions in a highly regulated manner resulting in effects that are beneficial for the bacteria. The pathogen *Yersinia* can resist phagocytosis by eukaryotic cells by translocating Yop effectors into the target cell cytoplasm. This is called antiphagocytosis, and constitutes an important virulence feature of this pathogen since it allows survival in immune cell rich lymphoid organs. We show here that the virulence protein YopK has a role in orchestrating effector translocation necessary for productive antiphagocytosis. We present data showing that YopK influences Yop effector translocation by modulating the ratio of the pore-forming proteins YopB and YopD in the target cell membrane. Further, we show that YopK that can interact with the translocators, is exposed inside target cells and binds to the eukaryotic signaling protein RACK1. This protein is engaged upon *Y. pseudotuberculosis*-mediated β1-integrin activation and localizes to phagocytic cups. Cells with downregulated RACK1 levels are protected from antiphagocytosis. This resistance is not due to altered levels of translocated antiphagocytic effectors, and cells with reduced levels of RACK1 are still sensitive to the later occurring cytotoxic effect caused by the Yop effectors. Further, a *yopK* mutant unable to bind RACK1 shows an avirulent phenotype during mouse infection, suggesting that RACK1 targeting by YopK is a requirement for virulence. Together, our data imply that the local event of *Yersinia*-mediated antiphagocytosis involves a step where YopK, by binding RACK1, ensures an immediate specific spatial delivery of antiphagocytic effectors leading to productive inhibition of phagocytosis.

## Introduction

The genus *Yersinia* contains three pathogenic species: *Yersinia pestis*, *Yersinia enterocolitica* and *Yersinia pseudotuberculosis* [1]. *Y. pestis*, the causative agent of plague, where bubonic plague is the most common form, is usually transmitted by the bite of a flea and spread to regional lymph nodes after which it can enter the bloodstream and spread systemically [2]. It is believed that *Y. pestis* replicate within macrophages during the early stages of infection at peripheral host sites [3], [4], [5], while extracellular growth is predominant during other stages of infection [6], [7]. The enteropathogenic species *Y. pseudotuberculosis* and *Y. enterocolitica* are transmitted by the oral-fecal route and penetrate the intestinal epithelium and spread to the lymphatic system (first Peyer's patches and thereafter mesenteric lymph nodes) where they replicate extracellularly [8], [9]. Also enteropathogenic *Yersinia* species can grow within macrophages [10], but if this occurs during infection, like for *Y. pestis*, is still controversal. Common to all three *Yersinia* species is their ability to replicate extracellularly in lymphoid tissue where most other bacteria are effectively engulfed and destroyed by phagocytes. Pathogenic *Yersinia* bind phagocytes and can impair their phagocytic capacity as well as certain inflammatory responses [11], [12], [13], [14], [15]. This permits bacterial survival and subsequent dissemination from these sites, resulting in systemic infection [16]. The capacity to survive and multiply in lymphoid tissue and to inhibit several important host immune mechanisms, including phagocytosis, is an essential virulence property of this pathogen.

The capacity of these three pathogenic *Yersinia* strains to multiply extracellularly and inhibit internalization by host cells depends on a virulence plasmid that encodes a common type three secretion system (T3SS) and virulence effectors such as the *Yersinia* outer proteins (Yops). The Yops include YopH, YopE, YopJ, YopM, YpkA, and YopK. Upon intimate contact with a target cell, these effectors are induced in the bacterium and delivered into the interacting host cell via a mechanism involving the plasmid encoded T3SS [17]. Inside the target cell, the Yop effectors interfere with several key mechanisms of the host immune defense including phagocytosis, production of pro-inflammatory signaling molecules, and activation of the adaptive immune system [18], [19]. Intracellular growth is however not dependent on the virulence plasmid [5]. Although most of the Yop effectors are necessary for *Yersinia* virulence, the exact mechanism underlying their individual roles is known only for a few [19]. For example, YopE is a Rho-GAP protein that mediates effects on the actin cytoskeleton and YopH is a tyrosine phosphatase that disrupts host cell signaling necessary for phagocytosis. This favours antiphagocytosis allowing bacteria to preferentially replicate extracellularly [13], [14], [20], [21], [22].

YopD, along with YopB and LcrV, is required for translocation of Yop effectors across the host cell plasma membrane. YopB and YopD contain hydrophobic domains indicative of transmembrane proteins and constituents of a pore [23], [24]. It is assumed that the Yop effectors pass through this pore when crossing the eukaryotic target cell membrane. Interestingly, *yopK* mutants form a larger pore and in line with this notion, *yopK* mutants overtranslocate Yop effectors [24].

YopK is one of the least studied of the translocated Yop effectors. This 21-kDa protein is found in all three of the human pathogenic species (i.e., *Y. pestis*, *Y. pseudotuberculosis*, and *Y. enterocolitica* (YopQ) [25], [26], [27], but it has no homologues in any other bacteria that harbor a T3SS. YopK has been shown to influence the size of the translocation pore [24] and is presumed to play a role in regulation of Yop effector translocation [24], [28]. This is supported by observations that a *yopK* deletion mutant generated larger pores and also over-delivered Yop effectors into target cells, whereas a strain overexpressing YopK reduced their translocation [24]. However, a *Y. pseudotuberculosis yopK* mutant was also avirulent in orally infected mice; bacteria were cleared at the stage of Peyer's patches, and never caused signs of systemic infection [25], [29]. Clearly therefore, YopK is essential for the ability of *Y. pseudotuberculosis* to cause full disease, via a hitherto unknown mechanism. Significantly, YopK is delivered into the target cell [30], which suggests that YopK has an intracellular target. In a recent report [31] it was suggested that YopK prevents inflammasome activation by the T3SS. This conclusion was based on the correlative observation that *yopK* mutants in contrast to wild-type bacteria activated the inflammasome. However, this observed response to infection with *yopK* mutants can equally well be explained by larger pores allowing influx/efflux of ions such as Ca^2+^ and K^+^ in addition to overtranslocation of effectors causing a stress response that activates the inflammasome.

The aim of the present study was to elucidate a possible YopK effector function inside the host cell. We identified the eukaryotic signaling protein called receptor for activated C kinase (RACK1) as a potential target of YopK. RACK1 is a cytosolic WD-40 repeat protein that was originally identified as being bound to and stabilizing the active form of protein kinase C (PKC) [32]. Additionally, RACK1 binds to β1-integrins [33], [34], [35], which also function as *Yersinia* receptors on the target cell surface [36]. We found that YopK is required for productive delivery of Yop effectors and, together with an interaction with RACK1, is crucial for *Yersinia* antiphagocytosis.

## Results

### YopK is associated with the translocation pore and is exposed to the host cell cytoplasm

**Figure 1:**
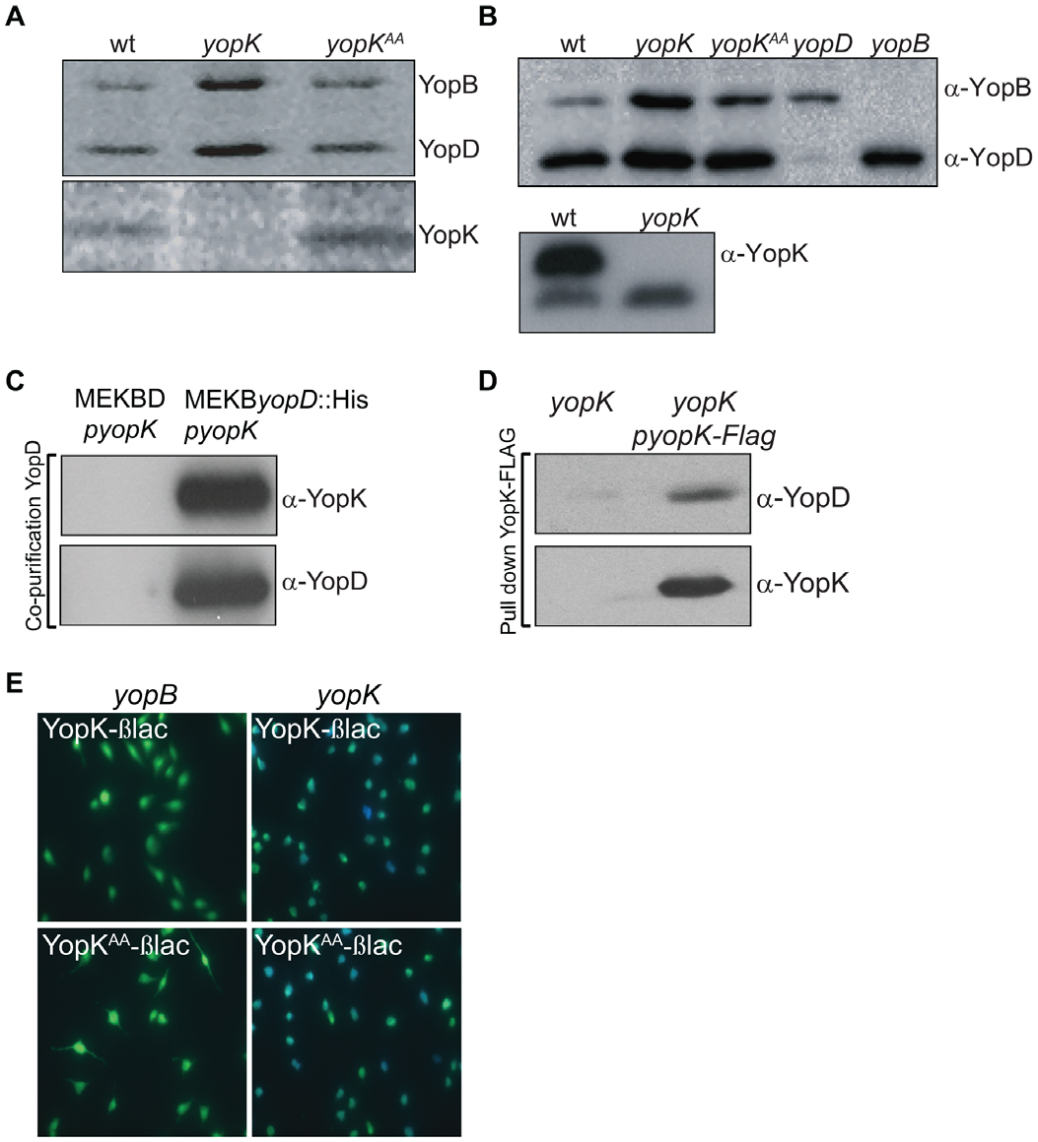
YopK interacts with translocation machinery components and is exposed inside target cells. (A) Autoradiogram showing presence of YopB, YopD, and YopK in purified membranes from erythrocytes infected with wild-type and isogenic mutants of *Y. pseudotuberculosis* labeled with ^35^S-methionine and ^35^S-cysteine. (B) Western blot showing YopB, YopD, and YopK in purified membranes from erythrocytes infected with wild-type and isogenic mutants of *Y. pseudotuberculosis*. The membrane fractions were separated on a 12% SDS page followed by Western blot analysis with antibodies raised against YopB, YopD and YopK. (C) Co-purification of YopD and YopK. His-YopD was purified from culture supernatants of a *Y. pseudotuberculosis* multiple *yop* mutant strain expressing His-YopD [YPIII(pIB29MEKByopD::His)]. Co-purification of YopK was detected by Western blot using antibodies against YopK. Detection of YopD by anti-YopD antisera was performed as a control. (D) Co-immunoprecipitation of YopK and YopD. Anti-FLAG beads were incubated with culture supernatants of a *Y. pseudotuberculosis yopK* mutant strain expressing YopK-FLAG *in trans*. Co-immunoprecipitation of YopD was detected by Western blot using antibodies against YopD. Detection of YopK by anti-YopK antisera was performed as a control. (E) Translocation of YopK–βlac and YopK^AA^–βlac into eukaryotic cells. HeLa cells were infected with the indicated *Y. pseudotuberculosis* strains, after which the FRET-based fluorescent β-lactamase substrate CCF2 was added and the samples were analyzed by fluorescence microscopy. The translocation-deficient *yopB* null mutant strain was used as negative control. Green cells indicate that no translocation occurred, whereas blue cells show that Yop–βlac fusion proteins were delivered into the HeLa cells. The percentage of blue cells, indicating translocation of YopK–βlac, was: 70+/−3.5% in the *yopK* mutant and 0% in the *yopB* mutant. The percentage of blue cells, indicating translocation of YopK^AA^–βlac, was: 58+/−5.2% in the *yopK* mutant and 0% in the *yopB* mutant. Data is presented as mean +/− SEM of at least six independent experiments.

Infection of erythrocytes is commonly used to study *Yersinia* pore formation. We have previously shown that hemolysis is induced to a much greater extent by a *yopK* null mutant of *Y. pseudotuberculosis* than by the corresponding isogenic wild-type strain [24]. The size of the bacterial translocation pore is also increased in the absence of YopK [24]. The *Y. pseudotuberculosis* translocation pore consists of the two hydrophobic translocator proteins YopB and YopD, which are inserted into the host cell membrane [37], [38], [39]. Interestingly, hemolytic assays in the presence of ^35^S-cysteine- or ^35^S-methionine followed by purification of red blood cell membranes and subsequent autoradiography revealed that YopK, together with YopB and YopD, was associated with the erythrocyte membranes (Figure 1A, lanes 1–2). The presence of these proteins, but not the other bacterial extracellular proteins (LcrV and the adhesin Ail), in the membrane fraction was further verified by Western blot analysis (Figure 1B and S1). Interestingly, quantification analysis revealed that the *yopK* null mutant inserted 4.4 times more of YopB (p = 0.04, n = 5) and 2.5 times more of YopD (p = 0,086, n = 5) in infected erythrocyte membranes compared to the wild-type bacteria. This increase in membrane associated translocators was not due to an increase in secretion, as the *yopK* null mutant secreted YopB and YopD to similar levels as the wild-type strain (data not shown). Importantly, the molar ratio between the two translocators determined by autoradiography was also altered: YopB/YopD was 0.71+/−0.025 for the mutant strain and 0.41+/−0.03 for the isogenic wild-type strain (Figure 1A). In addition, purification of a His-tagged YopD from bacterial supernatants resulted in co-purification of YopK (Figure 1C), and a reciprocal pull-down experiment using YopK–FLAG-coated beads showed co-precipitation of YopD (Figure 1D), thus confirming that YopK associates with the translocator proteins [31]. Furthermore, our data indicate that presence of YopK influences the pore composition where lack of YopK results in increased amounts of membrane associated translocator proteins resulting in a larger pore and as a consequence increased effector translocation.

Given that YopK could associate with the translocation pore, we also confirmed that it was exposed on the cytosolic side of the host plasma membrane. To address this, we used a fluorescence resonance energy transfer (FRET)-based β-lactamase reporter assay that was developed to discriminate between extra- and intracellularly localized T3SS effectors [40], [41]. Expression constructs encoding YopK–β-lactamase fusion proteins were generated and introduced into *yopK* and *yopB* null mutant strains; the latter strain was used as a translocation deficient control [23]. Using fluorescence microscopy we detected delivery of the YopK-βlac fusion protein into HeLa cells infected with a *yopK* null mutant (Figure 1E, upper right), but we did not detect delivery of the fusion protein into HeLa cells infected with the translocation-deficient *yopB* mutant (Figure 1E, upper left).

### YopK interacts with the host cell protein RACK1

**Figure 2:**
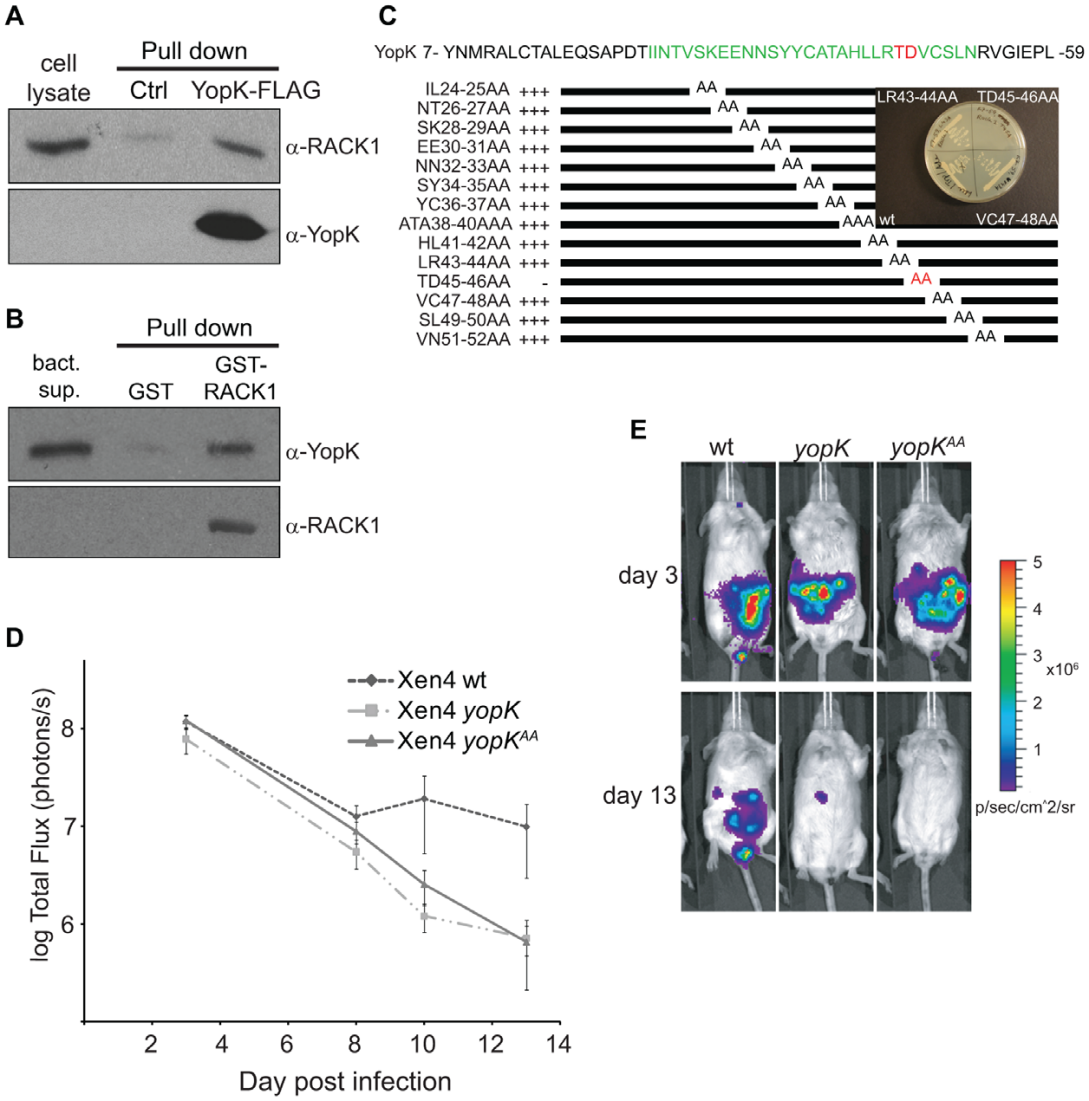
YopK interacts with RACK1 and this interaction is important for virulence. (A) Co-immunoprecipitation of YopK and RACK1. Beads with YopK-FLAG or control beads were incubated with HeLa cell lysates. Co-immunoprecipitation of RACK1 was detected by Western blot with anti-RACK1 antibodies. Detection of YopK by anti-YopK antisera was performed as a control, and HeLa cell lysate (12.5% input) was loaded for comparison. (B) Co-precipitation of purified recombinant RACK1 and YopK. Recombinant GST and GST-RACK1 on beads were incubated with culture supernatants of a *Y. pseudotuberculosis* multiple *yop* mutant strain expressing YopK-FLAG in trans [YPIII(pIB29MEKBD, pAH11)]. Co-precipitation of YopK was detected by Western blot with anti-YopK antibodies. Detection of RACK1 by anti-RACK1 antisera was performed as a control, and the bacterial culture supernatant (12.5% input) was loaded for comparison. (C) Pairwise alanine scan of YopK using the yeast two-hybrid assay as a read out. Interaction between the YopK variants and RACK1 was determined as growth of yeast on selective plates. In the diagram, +++ indicates growth corresponding to that seen in strains with non-mutated YopK and RACK1, and – stands for no growth. The mutated sequence is indicated in green and the amino acids identified as important for the interaction are in red. (D) Real-time monitoring of *Y. pseudotuberculosis* infection using the IVIS® technique. Mice were orally infected with 5.2×10^8^, 4.9×10^8^, and 6.3×10^8^ bacteria for the wild-type, *yopK*, and *yopK* ^AA^ strains respectively, and bioluminescent signals from the infected animals were plotted. Data are presented as photons/sec on the indicated days post infection, and the illustrated values represent mean ± sd for the five mice in each group. Data were compared by student's paired t-test, differences being considered significant at P<0.1. (E) Bioluminescent image of one representative mouse for each bacterial strain on days 3 and 13 post infection. The color bar presents the total number of emitted photons, with high and low bioluminescent signals indicated by red and blue, respectively.

Since YopK is indeed translocated, we next sought to identify a eukaryotic target protein by using YopK as bait in a yeast two-hybrid screen with a HeLa cell cDNA library. One of the positive clones identified encoded for the C-terminal portion (aa 71–317) of the scaffolding protein RACK1. This was particularly interesting since RACK1 interacts with the cytoplasmic domain of β-integrins [34] that serve as the receptors to which *Y. pseudotuberculosis* bind to establish contact with eukaryotic cells [36]. To verify that the YopK-RACK1 interaction observed in yeast also occurred in HeLa cells (our established eukaryotic cell model system), we performed pull down assays using an expression construct (YopK–FLAG fusion) and HeLa cell lysates. In short, secreted YopK protein was immobilized on beads, which were subsequently incubated with HeLa cell lysate. Western blot analysis of YopK-associated proteins revealed binding between RACK1 and YopK–FLAG (Figure 2A). This confirmed that YopK can interact with RACK1 in the eukaryotic cytosol, suggesting that this interaction is biologically relevant. To further explore the observed RACK1-YopK interaction, we performed additional pull down experiments, now using recombinant GST-RACK1 and supernatant from a multi-Yop mutant strain (unable to express YopE, YopH, YopM, YopB, YopD) expressing YopK. In brief, recombinant GST-RACK1 was immobilized on glutathione sepharose beads which were subsequently incubated with the *Yersinia* supernatant containing YopK. Western blot analysis of RACK1-associated proteins revealed binding between YopK and RACK1 (Figure 2B), thus indicating that the binding of YopK to RACK1 is direct, and not mediated by a bridging eukaryotic molecule.

To characterize the functional role of this interaction, we used the yeast two-hybrid system to map the respective interactive domains within both RACK1 and YopK. RACK1 forms a propeller-like structure that contains seven “blades” (domains 1 to 7), which consist of WD40 repeats that can bind to different ligands [42]. In our analysis, domains 5 and 6 of RACK1 proved to be sufficient for YopK binding (Figure S2A). Similarly, we generated and expressed different regions of YopK in the yeast system. This established that residues 30–59 were important for YopK to associate with RACK1 (Figure S2B). To further map the interaction, we performed a pair-wise alanine scan of the RACK1-binding region in YopK. This revealed that amino acids 45 and 46 in YopK were essential for binding to RACK1 (Figure 2C). Thus, we introduced these two amino acid changes (T45A, D46A) into the *yopK* gene *in cis* on the virulence plasmid pIB102 of *Y. pseudotuberculosis*, which resulted in the mutant strain YPIII(pIB156) (Table 1), hereafter denoted *yopK^AA^*. Phenotypic characterization revealed that this mutant was analogous to the *yopK* null mutant in key respects [24], [25]. Both strains maintained normal control of Yop expression and Yop secretion *in vitro* (data not shown) and the protease protection assay revealed that both strains translocated increased levels of Yop effectors into HeLa cells (Figure S2C). Further, YopK^AA^ was detected together with YopD and YopB in the plasma membrane of infected erythrocytes (Figure 1A, lane 3) and located to the cytosolic side of the HeLa cell membrane (Figure 1D, bottom left and right). Moreover, similar to the *yop*K mutant (described above), infection with the YopK^AA^-expressing strain also resulted in larger amounts of especially the translocator YopB (1.5 times more) in the target cell membrane resulting in an altered YopB/YopD ratio (0.52+/−0.04) compared to that seen with the wild-type strain (0.41+/−0.03). The observed effect on the translocator amount and ratio was however less prominent than that seen with the *yopK* mutant, which likely depends on presence of the mutated YopK^AA^.

**Table 1:** *Y. pseudotuberculosis* strains and plasmids used in this study.

Strain	Relevant genotype	Source or Reference
*Yersinia pseudotuberculosis*		
YPIII	Virulence plasmid cured	[76]
YPIII(pIB102)	*yadA*, Km^R^ (wild-type)	[62]
YPIII(pIB155)	*yadA, yopK*, Km^R^	[25]
YPIII(pIB156)	*yadA, yopK _T45A, D46A_*, Km^R^	This study
YPIII(pIB526)	*yadA, yopE,* Km^R^	[28]
YPIII(pIB604)	*yadA*, *yopB*, Km^R^	[23]
YPIII(pIB621)	*yadA, yopD,* Km^R^	[66]
YPIII(pIB29MEKB)	*yadA, yopH, yopM, yopE, yopK, yopB, ypkA*, Km^R^	[23]
YPIII(pIB29MEKBD)	*yadA, yopH, yopM, yopE, yopK, yopB, yopD, ypkA,* Km^R^	This study
YPIII(pIB29MEKByopD::His)	*yadA, yopH, yopM, yopE, yopK, yopB, ypkA, yopD::his*, Km^R^	This study
YPIII(pCD1, Xen4)	*Tn1000::Tn5 luxCDABE,* Km^R^	Caliper Life Sciences
YPIII(pCD155, Xen4)	*Tn1000::Tn5 luxCDABE*, *yopK*, Km^R^	This study
YPIII(pCD156, Xen4)	*Tn1000::Tn5 luxCDABE*, *yopK_T45A, D46A_*, Km^R^	This study
**Plasmids**		
pAH3	*yopK^+^,* Amp^R^	[25]
pTS103	*exoS^+^*, Amp^R^	[77]
pYopK-βlac	*yopK-*β*lac^+^,* Cml^R^	This study
pYopK^AA^-βlac	*yopK-*β*lac^+^*, Cml^R^	This study
pAH11	*yopK-Flag^+^,* Amp^R^	(A. Holmstrom, unpublished)
pMF115	*yopK^+^,* Amp^R^	This study
pGEX-RACK1	*Gst-Rack1^+^,* Amp^R^	This study

To gain further insight into the importance of YopK and the interaction with RACK1 for *Y. pseudotuberculosis* virulence, we took advantage of the new *in vivo* imaging system technology that allows real-time monitoring of *Yersinia* infections [43]. The *yopK* deletion and *yopK^AA^* substitution were introduced into the bioluminescent *Y. pseudotuberculosis* wild-type strain YPIII(pCD1 Xen4). Female BALB/c mice were orally infected with 5.2×10^8^, 4.9×10^8^, and 6.3×10^8^ bacteria for the wild-type, *yopK*, and *yopK* ^AA^ strains, respectively. During the course of infection, mice were visualized daily and analyzed for bioluminescence on days 3, 8, 10, and 13. All mice infected with wild-type bacteria showed clear signs of disease (ruffled fur and diarrhea) on days 4–6, whereas mice infected with either of the *yopK* mutant strains were apparently healthy. Analysis of total light emitted from the infected mice revealed that all were similarly colonized by the three *Yersinia* strains during the first eight days. Thereafter, the signals from mice infected with either of the two *yopK* mutant strains declined, while signals from animals infected with the wild-type strain remained constant (Figures 2D and 2E), showing a significant difference between the signals from mice infected with the wild-type strain compared to the two *yopK* mutants. Thus, the two *yopK* mutants are similarly also attenuated during mouse infection.

### *Yersinia* antiphagocytosis requires RACK1

**Figure 3:**
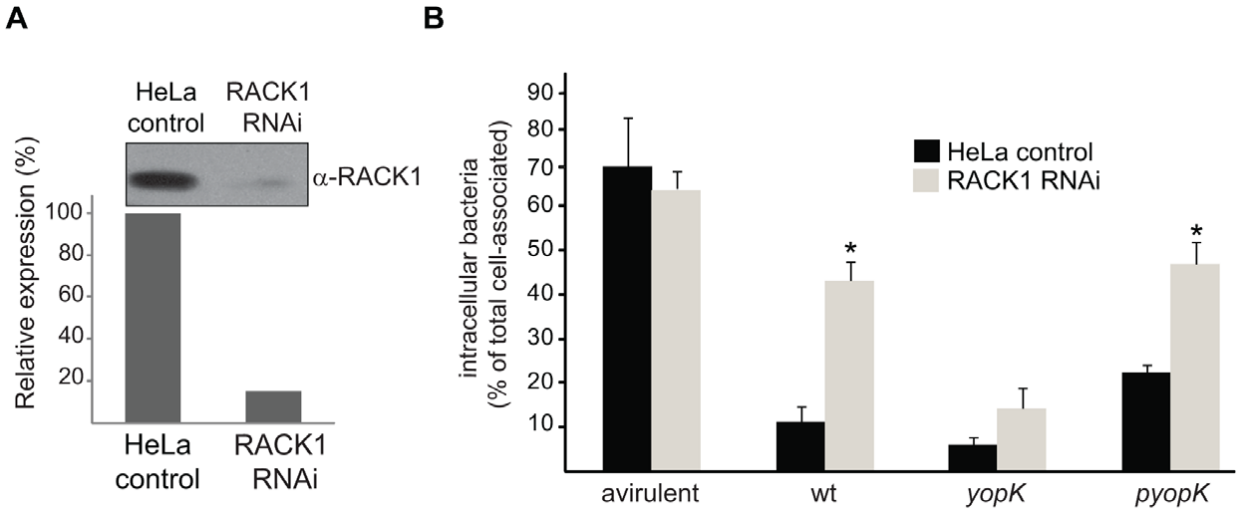
RACK1 is required for antiphagocytosis. (A) Downregulation of RACK1 protein expression in HeLa cells by RNAi. Lysates of control and RACK1 RNAi HeLa cells analyzed by Western blot using anti-RACK1 antibodies. Levels of RACK1 protein are presented as expression relative to that in HeLa control cells. (B) Internalization of the indicated *Y. pseudotuberculosis* strains by control and RACK1 RNAi HeLa cells. The number of bacteria internalized is presented as percent of the total number associated with HeLa cells. The illustrated data represent mean ± SEM of at least three independent experiments (P<0.05 *).

RACK1 is a ubiquitously expressed protein that interacts with a large array of signaling molecules, regulating cellular functions such as adhesion, movement, and division [42]. In particular, RACK1 interacts with the cytoplasmic domain of β1-integrins [34] and was also recently reported to bind focal adhesion kinase and participate in signaling from adhesion receptors [44]. Given that β1-integrins constitute the eukaryotic cell receptors to which *Yersinia pseudotuberculosis* docks via its adhesin invasion [36], perhaps RACK1 participates in the bacterium-induced β1-integrin-mediated events. This idea was appealing because it could also mean that YopK binds to RACK1 to directly interfere with a signaling pathway that is important for host cell defense against the pathogen. To test this idea, we exposed HeLa cells to lentivirus-mediated RNAi of RACK1 to generate stable cell lines with downregulated RACK1 expression. A stable clone with 85% reduction of RACK1 expression (Figure 3A) (hereafter denoted RACK1 RNAi cells) was selected for further investigation.

An essential virulence feature of both enteropathogenic yersiniae and *Y. pestis* is the ability to block phagocytic uptake to avoid mechanisms of the innate host defense. Inhibition of internalization of the bacteria by eukaryotic cells has been demonstrated using professional phagocytes and was initially designated antiphagocytosis [11], [13], [15], [45]. Since the underlying mechanism of inhibition of internalization is similar in phagocytes and HeLa cells, the term antiphagocytosis is hereafter used for defining inhibition of bacterial internalization by both cell types (the term does not include the virulence plasmid independent avoidance of intracellular killing [4]). Non-opsonised *Y. pseudotuberculosis* is mainly internalized via the invasin-β1-integrin interaction in both these cell types [36], [45], [46], [47]. To ascertain whether RACK1 is involved in phagocytosis of *Y. pseudotuberculosis* or if it interferes with antiphagocytosis, we performed infection experiments using RACK1 RNAi cells and determined the amount of extracellular and intracellular bacteria. In agreement with previously reported results [13], the plasmid-cured avirulent *Y. pseudotuberculosis* strain YPIII was efficiently internalized by control HeLa cells (69% internalization) (Figure 3B), whereas the corresponding wild-type strain remained largely extracellular (11% internalization) (Figure 3B). Furthermore, the avirulent YPIII strain was still efficiently phagocytosed by the RACK1 RNAi cells (64% compared to 69% internalization by the HeLa control cells) (Figure 3B). Hence, eukaryotic cells do not require RACK1 to internalize *Yersinia*. Surprisingly, the virulent wild-type strain displayed a significant reduction in the ability to block phagocytosis by RACK1 RNAi cells (43% internalization) (Figure 3B). Analyses of bacterial adherence by counting total number of cell-associated bacteria revealed that the bacteria adhered to the RACK1 RNAi cells to similar extent as to the control cells. This implies that in the absence of RACK1, eukaryotic cells are protected from the *Yersinia* antiphagocytic attack. Notably, the absence of RACK1 could not protect RACK RNAi cells from antiphagocytosis induced by the *yopK* null mutant or the *yopK^AA^* mutant (Figure 3B and data not shown). In contrast, RACK1 RNAi cells were resistant towards the antiphagocytic activity of a *trans*-complemented *yopK* null mutant (Figure 3B). While this might mean that YopK itself is an effector protein directly involved in the antiphagocytic mechanism, we could not find any evidence in support of this. Isogenic *yopK^+^* and *yopK^−^* mutants (both unable to express YopH, YopE, YopM, and YpkA) were indistinguishable from each other with respect to their ability to block phagocytosis in HeLa cells that were or were not expressing RACK1 (data not shown). Since YopK and RACK1 interact, a plausible interpretation of these surprising findings is that in the absence of RACK1, YopK has a negative impact on translocation control, either at the level of effector delivery or effector targeting.

To confirm that the reduced antiphagocytic effect on RACK1 RNAi cells was actually due to the presence of lower levels of RACK1, and not an indirect effect of clonal selection or the RNAi procedure itself, we analyzed an additional RACK1 RNAi cell line with approximately similar level of RACK1 downregulation, but obtained with an oligo directed to another target sequence. Also this RACK1 RNAi cell line was resistant towards antiphagocytosis (Fig S3A), thus excluding an eventual off-target effect. In addition, we included two other RACK1 RNAi cell lines in the analysis; one that displayed 60% downregulation of RACK1, while the second was not downregulated at all. Similar levels of antiphagocytosis were achieved by the wild-type strain in the cell line with undetectable RACK1 downregulation and the control HeLa cells (Figure S3B). On the other hand, the extent of antiphagocytic activity was intermediate in the cells with 60% RACK1 downregulation compared to the level in cells with 85% downregulation (Figure S3B). Thus, there was a direct dose-response correlation between the level of bacterial phagocytosis and the amount of RACK1. Hence, together these experiments show that the observed reduction in antiphagocytosis is linked to low levels of RACK1 in the target cells.

### Translocation of Yop effectors does not require RACK1

**Figure 4:**
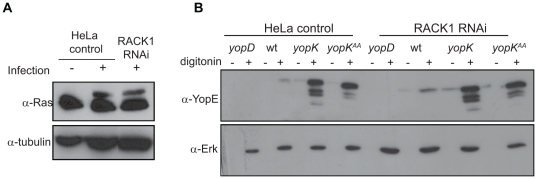
RACK1 is not required for Yop translocation. (A) Translocation of ExoS resulting in modification of Ras in control and RACK1 RNAi HeLa cells. The cells were infected with a *Y. pseudotuberculosis* strain expressing ExoS [YPIII(pIB526), pTS103]. Modification of Ras was visualized by Western blot with anti-Ras antibodies. Detection of tubulin was used as loading control. (B) Translocation of YopE into control and RACK1 RNAi HeLa cells. Cells were infected with the indicated strains and then treated with proteinase K. Thereafter, the cells were lysed with digitonin or left untreated. The resulting supernatants were subjected to Western blot analysis using anti-YopE antisera. The translocation deficient *yopD* mutant was included as negative control and detection of Erk was used as loading control.

To determine whether the level of RACK1 influences the amount of Yop effectors translocated, we employed two different methods to analyze T3SS-mediated translocation of effectors into eukaryotic cells: a translocation assay using the *Pseudomonas aeruginosa* effector ExoS [48] and the protease protection assay [49]. In the former technique, the ExoS-dependent ADP ribosylation of cytoplasmic proteins (e.g., Ras) allows sensitive detection of ExoS translocation into eukaryotic cells. We used the ExoS-producing *Y. pseudotuberculosis* strain YPIII(pIB526pTS103) (Table 1) to infect control and RACK1 RNAi HeLa cells. Lysates were analyzed by Western blot to detect modification of Ras. Both cell lines displayed comparable levels of Ras modification, indicating that similar amounts of ExoS were translocated into the different cells (Figure 4A). This agreed with the results of the protease protection assay where similar levels of the antiphagocytic effector YopE were translocated into the two cell types (Figure 4B). Collectively, this implies that RACK1 does not affect the level of Yop effector translocation *per se*. Consequently, the reduced antiphagocytic capacity of *Y. pseudotuberculosis* in contact with RACK1 RNAi cells cannot be explained by altered levels of antiphagocytic effectors inside these cells.

### *Yersinia*-mediated cytotoxicity does not require RACK1

Secondary to antiphagocytosis, prolonged infection of adherent cells induces cell rounding, which is often followed by detachment from the underlying substratum. This is due to YopE-mediated disruption of F-actin organization, an effect that is referred to as cytotoxicity [50]. This cytotoxicity assay is often employed to evaluate the general effects of Yop effector function. We used the assay here to determine whether the YopK–RACK1 interaction is important also for this late and more devastating impact of prolonged exposure to the antiphagocytic effectors. Interestingly, the RACK1 RNAi cells and the control HeLa cells were equally affected by the cytotoxic capacity of the *Y. pseudotuberculosis* wild-type strain and also by the *yopK* null and *yopK^AA^* mutant strains (Table 2). Hence, the absence of RACK1, which protects cells from the immediate effects of antiphagocytosis, does not protect against the late onset global cytotoxic effects mediated by prolonged exposure to the bacteria. This shows that the YopK–RACK1 interaction is exclusively needed for the immediate local effect in close proximity to the bacterial attachment site, which represents the inhibitory influence of antiphagocytic effectors.

**Table 2:** *Yersinia* mediated cytotoxicity does not require RACK1.

Strain	Cytotoxicity in HeLa control cells	Cytotoxicity in RACK1 RNAi cells
avirulent	−	−
wt	++	++
*yopK*	+++	+++
*yopK^AA^*	+++	+++

### RACK1 localizes to phagocytic cups

**Figure 5:**
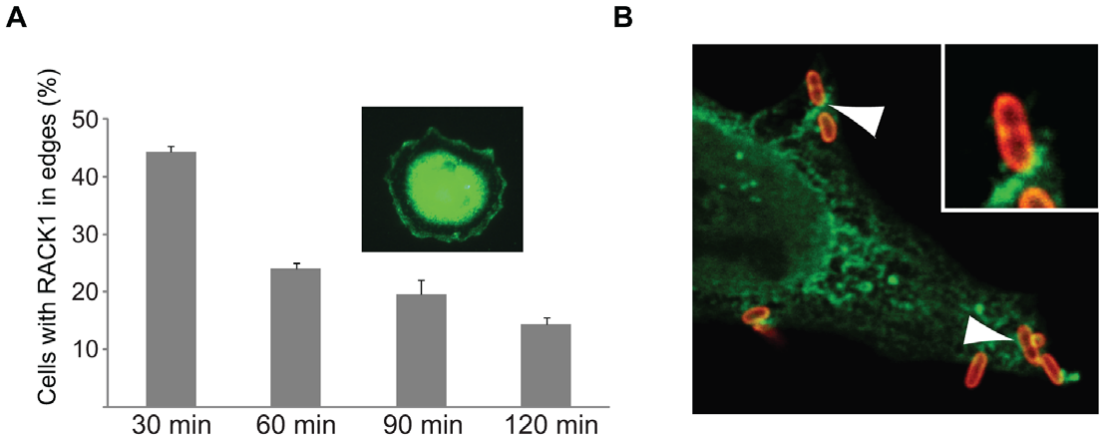
RACK1 localizes to phagocytic cups in infected HeLa cells. (A) RACK1 is recruited to the edges of HeLa cells upon β1-integrin activation. HeLa cells were allowed to spread on coverslips coated with the β1-integrin ligand invasin. At indicated time points, cells were fixed, stained for RACK1, and analyzed by immunofluorescence microscopy. The number of cells with RACK1 localized in the edges is presented as percent of the total number of cells. The data represent mean ± SEM of at least three independent experiments. The inset shows a representative cell at 30 min. (B) Localization of RACK1 in HeLa cells during infection with *Y. pseudotuberculosis*. HeLa cells were infected for 5 minutes, fixed and stained for RACK1 (green) and *Yersinia* (red). The samples were analyzed by confocal microscopy.

Our finding that the RACK1–YopK interaction is a local event selectively important for antiphagocytosis prompted us to investigate the involvement of RACK1 in host cell signaling induced by cell surface binding of the bacteria. Upon infection, *Y. pseudotuberculosis* bind to, cluster, and activate β1-integrins [36], resulting in internalization of the bacteria in the absence of Yop effectors. Given that RACK1 interacts with the cytoplasmic part of β1-integrins and with associated signaling proteins [34], [44] it could possibly participate in signaling mechanisms activated by the bacterial binding. To test this, we analyzed the subcellular localization of RACK1 during β1-integrin-mediated cell spreading. In the spreading assay, cells were detached and allowed to rest before exposure to a surface coated with *Y. pseudotuberculosis* invasin encompassing the β1-integrin-binding region [51]. Interestingly, while RACK1 was localized to the perinuclear area in routinely cultured HeLa cells (Figure S4), a fraction of RACK1 was transiently recruited to the cell edges during β1-integrin stimulated cell spreading (Figure 5A). This was most prominent after 30 min incubation on the invasin-coated surface, by which time most of the cells had started to spread. Thus, RACK1 is recruited to areas of β1-integrin activation during initial spreading, which suggests that RACK1 participates in the early events of β1-integrin signaling. This is in agreement with a previous study suggesting that RACK1 can control initiation of cell spreading [44]. Consequently, it is likely that an interaction between the bacterium and the host cell induces a corresponding recruitment of RACK1 to the bacterial binding site. To test this, we performed immunofluorescence analysis to visualize the localization of RACK1 in HeLa cells internalizing *Y. pseudotuberculosis*. In support of our hypothesis, RACK1 was found in association with phagocytic cups, where the bacteria were partially surrounded by the HeLa cell membrane (Figure 5B). This implies that RACK1 is engaged during β1-integrin-mediated internalization of *Yersinia*, and is recruited to intracellular areas that are near an extracellularly located bacterium. Together with our data showing that YopK is connected to the translocation complex and exposed on the cytosolic side of the host cell plasma membrane, the data collectively strengthen our hypothesis that YopK targets RACK1 in the host cell.

## Discussion

Pathogenic *Yersinia* species utilize a powerful T3SS to deliver effector proteins into the interior of the targeted host cells [17]. A set of translocator proteins form a pore in the host cell membrane through which these effectors are thought to gain access into the host cell cytosol. This virulence mechanism allows these bacteria to purposefully block innate immune cell defense mechanisms such as phagocytosis so they can proliferate in lymphatic tissue [13]. However, the phagocytic process is initiated as soon as a bacterium comes in contact with a target cell and completed within minutes after cell contact. Therefore, it is assumed that pathogenic *Yersinia* battle to impair this process via a very rapid and precise mechanism for effector delivery and action. In line with this, we have previously shown that Yop effector deployment and intracellular activity can be measured within 30 seconds after the bacterium makes contact with a host cell [45], [52]. Nevertheless, extreme rapidity alone would not be sufficient; individual Yops would also need targeting to the precise site of action. Regulation of this process in the bacterium occurs on several levels; this includes coordinating target cell contact with induction of gene expression [53], subsequent feed-back repression [28], and via control of effector translocation [24]. We have presented data in this study to support a mechanism that guarantees productive antiphagocytic effector translocation leading to an instant blockage of phagocytosis. It relies upon an interaction in the host cell between YopK, which is associated with the pore complex, and the eukaryotic protein RACK1.

We observed that RACK1 was specifically required for antiphagocytosis. This was based on the finding that host cells downregulated for RACK1 expression engulfed the pathogen to a much greater extent compared to cells with normal RACK1 expression, implying that these RACK1 RNAi cells are resistant to the antiphagocytic activity of the T3SS. This was quite unexpected; making it the first reported cell line to be impervious to the action of the *Yersinia* antiphagocytic machinery. Importantly, virulence effectors were translocated to the same extent into cells regardless of their RACK1 expression level and these intracellular effectors still induced a normal cytotoxic response in both cell lines. This means two things; firstly, translocation of effectors and the process of cytotoxicity induction do not require RACK1, and secondly, antiphagocytosis and cytotoxicity are clearly two distinct events during cell infection. Another striking feature was that the ability of the pathogen to mediate antiphagocytosis towards RACK1 RNAi cells diminished significantly when YopK was present. Notably, this failure to inactivate the critical cellular targets and interrupt phagocytosis occurred despite efficient antiphagocytic Yop effector translocation into the target cells. Hence, the discrepancy between *Yersinia* YopK^+^ bacteria inducing an uncompromising cytotoxic response on the one hand, but failing to establish antiphagocytosis on the other, suggests that the latter process requires specific features of the effector translocation process that are overseen by YopK. This agrees with our previous results concerning the antiphagocytic effector and major cytotoxin YopE [54]; certain *yopE* point mutants exhibited attenuated virulence but still gave rise to cytotoxicity in HeLa cells. Thus, the cytotoxic effect is actually a secondary event that occurs only after prolonged infection and is dispensable for *in vivo* virulence. On the other hand, there appears to be a direct correlation between the ability of *Y. pseudotuberculosis* to resist phagocytosis and to cause systemic infections in mice. This is based on the fact that except translocation-deregulated *yopK* mutants, no other attenuated Yop effector mutant possesses full antiphagocytic capacity. In fact, mutants defective in antiphagocytosis are cleared at the initial stage of infection in Peyer's patches and mesenteric lymph nodes [25], [29].

YopK and antiphagocytosis are inextricably linked. For several years, YopK has been suspected to affect the Yop translocation pore [24]. Herein, we show that YopK localized with the translocators YopB and YopD in the membrane fraction of infected cells. In addition, YopK can be found inside infected host cells – presumably at the zone of bacteria-host cell contact. Interestingly, we observed that RACK1 also accumulates at the site of bacterial attachment to host cells and immediately upon activation of β1-integrins. We believe that this RACK1 localization is a prerequisite for productive blocking of phagocytosis since such a location would enable RACK1 to interact with YopK associated with the T3SS. Indeed, using independent protein-protein binding assays, we could demonstrate this interaction. Together, these data suggest that YopK functions as a sensor and mediator of productive effector translocation. Thus, it is feasible that the YopK-RACK1 binding contributes to efficient antiphagocytosis where the association of YopK with the translocation pore ensures that translocated effector proteins are immediately exposed to their key target(s) represented by signaling proteins involved in β1-integrin-mediated internalization. Interesting in this context is that RACK1, located at peripheral initial adhesion structures, has been shown to bind to focal adhesion kinase [44], which constitutes a target of the antiphagocytic effector YopH [55].

**Figure 6:**
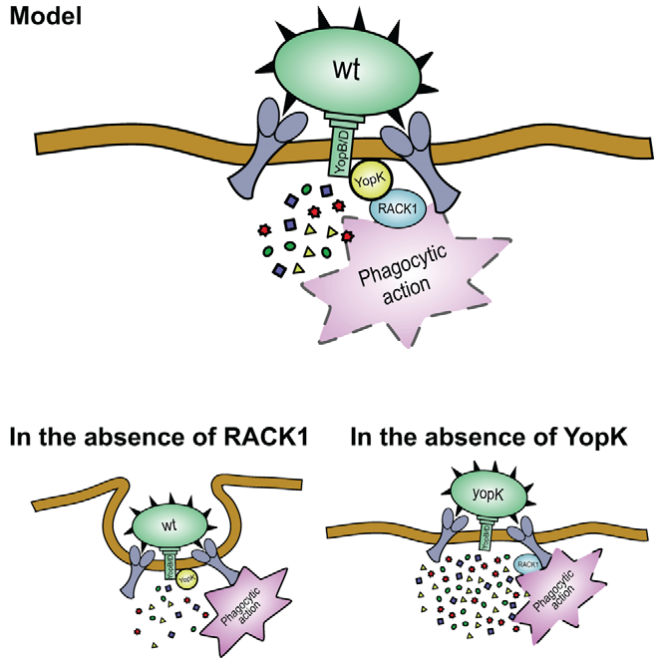
Hypothetical model illustrating how *Y. pseudotuberculosis* utilizes RACK1 for productive translocation resulting in anti-phagocytosis. Upon host cell contact, bacterial binding to β1-integrins causes clustering and activation of this receptor. This leads to engagement of signaling proteins in the host cell, including RACK1 that is recruited to the zone of bacteria-host cell contact. At this site of action, RACK1 functions as a marker for an activated phagocytic signaling machinery that is specifically recognized by YopK. Binding of RACK1 by YopK orchestrates correct temporal and spatial programming of Yop delivery to concentrate internalized antiphagocytic effectors at the “right place” where they instantly can bind to and inactivate their protein target(s). This permits *Yersinia* to rapidly neutralize the very early signals originating from activated receptors and thereby ensure instant impairment of the phagocytic process. In the absence of RACK1 (RACK1 RNAi cells), the spatial control of effector translocation is lost resulting in aimless Yop delivery. This diminishes a critical effector concentration in the zone of bacteria-host cell contact that would normally be needed to effectively negate phagocytic signaling. This leads to a non-productive interaction between the pathogen and the target cell that terminates in bacterial engulfment. In the absence of YopK (*yopK* mutant) the translocation of Yops is deregulated resulting in excessive delivery of Yop effectors. Thus, although Yop effector delivery would be aimless due to the absence of the YopK-RACK1 interaction, the over-supply of available Yops ending up inside the target cell will ensure that a critical concentration necessary to dismantle phagocytic targets within the host cell would still be reached. However, still *yopK* mutants are avirulent, indicating that surplus delivery of effectors is clearly detrimental for bacterial survival *in vivo*.

From this work, we suggest that RACK1 serves as a recognition site for YopK to obtain a precise spatial translocation of antiphagocytic effectors allowing instant targeting of the phagocytic machinery. At first glance however, this might be difficult to reconcile considering that antiphagocytosis works fine in the absence of YopK, or both YopK and RACK1, but not in the absence of RACK1. In this respect, consider the critical finding that RACK1 RNAi cells resist the antiphagocytic effect of wild type *Y. pseudotuberculosis*, but not of the *yopK* mutants. Thus, the key to unlocking this conundrum is to understand how *yopK* mutants suppress the RACK1 minus phenotype. The most likely reason for this is that these mutants show an overtranslocation phenotype, resulting in excessive delivery of Yop effectors into the host cell [24]. Although effector delivery in the absence of YopK would be random and unorganized, their shear excess inside the cell will ensure that they still find and inactivate the critical target(s) within the necessary time-frame. Therefore, this excess of effectors would overcome any need for YopK-RACK1 recognition; after all, only in the presence of YopK is RACK1 required for antiphagocytosis! Consistent with this is the avirulent phenotype of *yopK* mutants; surplus delivery of effectors is clearly detrimental for bacterial survival *in vivo*. The *yopK* mutants were cleared after a week of infection, likely as a consequence of damaging effects on host cells resulting in increased recruitment of immune cells and/or inundating the host with antigenic epitopes against which it can trigger a more robust and effective immune response. These mutants might also display reduced *in vivo* fitness because of the high energetic cost associated with Yop effector over-translocation. Whatever the reason(s), it suggests that RACK1 targeting by YopK is a key step in the controlled, target-directed process of effector delivery and a requirement for virulence. Antiphagocytosis by *Yersinia* is immediate, which contrasts to other pathogenic bacteria harboring a T3SS such as Enteropathogenic *E. coli* (EPEC) and Enterohemorrhagic *E. coli* (EHEC) that can induce an antiphagocytosis-like response after prolonged bacteria-host cell exposure [56], [57], [58], [59]. Since YopK is unique to the *Yersinia* T3SS, we argue that its interaction with RACK1 forms the basis for the immediate blocking of phagocytosis, a hallmark of this pathogen. We suggest that this also applies for *Y. pestis* during the extracellular stages of infection. *Y. pestis* do not express a functional invasin, but the adhesins Ail and Pla bind the extracellular matrix protein fibronectin that can interact with β1-integrins [60]. This pathogen that is more virulent than the enteropathogenic strains and exhibits an extremely efficient growth in lymph nodes during late phases of infection is also equipped with an antiphagocytic capsule, which likely contributes [15], [61]. Taken together, based on our data, we suggest a hypothetical model of this YopK-RACK1 interplay that would account for the ability of *Yersinia* to cause an instant phagocytic block (Figure 6). In this model, antiphagocytosis involves action at a distance from the bacterial surface where YopK ensures specific spatial delivery of antiphagocytic effectors using RACK1 as a marker for an active phagocytic signaling machinery.

## Materials and Methods

### Ethics Statement

Mice were housed under standard conditions and were given food and water ad libitum. The experiment was approved (Dnr A81-08) by, and performed according to the guidelines of, the Umeå University Animal Ethics Committee.

### Bacterial strains and growth conditions

This study was conducted using wild-type *Y. pseudotuberculosis* YPIII(pIB102) [62] harboring the 70 kb virulence plasmid and isogenic mutant strains thereof (listed in Table 1). Before all experiments, bacteria were grown overnight at 26°C in Luria-Bertani broth (LB), TMH, or Brain Heart Infusion (BHI) supplemented with 5 mM EGTA and 20 mM MgCl_2_. To induce Yop expression, the bacteria were diluted in LB, TMH, BHI, or antibiotic-free cell culture medium and grown at 26°C for 30 min and subsequently at 37°C for 1–3 hr.

### Plasmid construction

To generate the YopK–GAL4-BD fusion construct pMF115, the *yopK* gene from the *Y. pseudotuberculosis* virulence plasmid pIB102 was amplified by PCR and cloned into the EcoRI/PstI sites of the yeast plasmid pGBT9 (Clontech Laboratories Inc). The *yopK–Flag* expression vector was obtained by constructing an in-frame carboxy-terminal fusion of YopK, and the eight-amino-acid FLAG epitope DYKDDDDK [63] by PCR amplification using the virulence plasmid pIB1 as template. The 972-bp fragment that contained the *yopK* gene with the FLAG epitope and 426 bp of upstream DNA was cloned into the EcoR1/XbaI sites of pUC19. The resulting plasmid (pAH11) was introduced into appropriate *Y. pseudotuberculosis* strains by electroporation.

To obtain fusion proteins between full-length YopK/YopK^AA^ and β-lactamase lacking its N-terminal signal peptide, PCR amplifications and standard molecular methods were performed. First a DNA fragment corresponding to amino acids 24–286 of the β-lactamase gene from the plasmid pTK-Hyg (Clontech Laboratories Inc) was cloned into the HindIII/XbaI sites of pMF353, a modified version of the pPROLar.A expression vector (Clontech Laboratories Inc) in which the SacI/AatII DNA fragment containing the kanamycin resistance gene had been exchanged for the chloramphenicol resistance gene from the pPROTet.E vector (Clontech Laboratories Inc). Then, full length *yopK* or *yopK^AA^* sequences were PCR amplified from the plasmids pIB102 and pIB156 (described below) and cloned in frame with the β-lactamase gene using the upstream KpnI/HindIII sites.

The *Y. pseudotuberculosis yopK^AA^* mutant strain YPIII(pIB156) was generated by homologous recombination. The point mutations were obtained using an overlapping PCR strategy described in detail elsewhere [64]. A 600 bp DNA fragment covering the *yopK* T45A-D46A substitution in the center was cloned into to the suicide vector pDM4. The T45A-D46A mutation was transferred from the suicide vector down to the virulence plasmid by conjugation and double cross-over performed using previously reported methodology [65]. Similarly, to generate the *Y. pseudotuberculosis* multiple *yop* mutant YPIII(pIB29MEKBD), the pDM4-derived mutagenesis vector pMF024 [66] was conjugated into YPIII(pIB29MEKB) [23] and the full length *yopD* deletion introduced onto the virulence plasmid by allelic exchange.

The GST-RACK1 expression construct was generated by PCR amplification of the RACK1 fragment from the eukaryotic expression vector pEBG-RACK1 (kindly provided by Dr Chang [34]) and cloned into the pGEX-4T-1vector (GE Healthcare).

### Yeast two-hybrid screen

Analysis of protein–protein interactions was performed according to the MATCHMAKER protocol (Clontech Laboratories). The pGBT9 encoding YopK fused to the GAL4 DNA-binding domain was transformed into *Saccharomyces cerevisiae* strain PJ69-4A [67], and this strain was then used as recipient in transformations with a pACT2-based Human HeLa MATCHMAKER cDNA library (Clontech Laboratories). An interaction between YopK and a protein encoded by cDNA from the HeLa cell library was determined as growth of yeast at 30°C on minimal medium lacking adenine, tryptophan, and leucine. To confirm that the growth on selective plates was due to this interaction, yeast was cured of one or both plasmids and re-assayed.

### Mapping the YopK-RACK1 interaction

Different regions of the *yop*K gene (indicated in Figure S2A) were amplified by PCR and cloned into the *Eco*RI/*Pst*I sites of pGBT9 (Clontech Laboratories) to create in-frame fusions between different parts of YopK and the GAL4 DNA-binding domain. The resulting fusions were tested for interaction with the isolated pACT-RACK1_71-317_ clone identified in the initial screen. Amino acids 30–59 of YopK were identified as a RACK1-binding region, which was further analyzed by introducing pair-wise alanine substitutions covering aa 26–52 (indicated in Figure 1A) in the truncated aa 7–59 form of the protein by use of a two-step PCR strategy and synthetic oligonucleotides, as previously described [64]. The mutations were verified by sequencing, and all substitution mutants were subsequently tested in the yeast two-hybrid assay for interaction with RACK1, as described above.

### Detection of YopB, YopD, and YopK in erythrocyte membranes

Contact-dependent hemolysis of sheep erythrocytes was carried out as previously described [23], [24], [37], [68]. Radiolabeling hemolytic experiments were performed in TMH with 0.1 mM methionine supplemented with ^35^S-labeled methionine (YopB/YopD) or ^35^S-cysteine (YopK), in 2 ml reactions containing 1.5×10^8^ bacteria, and 4×10^9^ washed sheep erythrocytes (National Veterinary Institute Sweden). The hemolytic assay mixtures were supplemented with 2 mM DTT when ^35^S cysteine was used. Isolation of erythrocyte plasma membranes was achieved by density centrifugation as previously described [69], [70] but using an additional step involving low-intensity sonication of the bacteria-free lysate to increase the recovery of the erythrocyte membranes. The protein concentration of the membrane preparations was determined by measurements at OD_280_ and equal amounts of purified membrane proteins were separated by SDS-PAGE. The labeled proteins were visualized by exposing dried Coomassie-stained gels on a storage phosphor screen (Molecular Dynamics) and then developing in a Storm 860 imaging system (Molecular Dynamics). The proteins were quantified using the Quantity One software package (Bio-Rad). The presence of the proteins was further verified by Western blot analysis of separated membrane fractions.

### Cell lines

HeLa cells (DSMZ) were maintained in Minimum Essential Medium (MEM) (Sigma) supplemented with 10% fetal calf serum (PromoCell), 2 mM GlutaMAX^TM^-I (Invitrogen), 100 U/ml penicillin, and 100 µg/ml streptomycin (Invitrogen). For virus production, human 293FT cells (Invitrogen) were maintained in Dulbecco's Modified Eagle's Medium (DMEM) (Gibco) supplemented with 10% fetal bovine serum (Invitrogen), 0.1 mM MEM Non-Essential Amino Acids (Invitrogen), and 500 µg/ml Geneticin® (Invitrogen). All cell lines were cultivated at 37°C in a humidified atmosphere containing 5% CO_2_. For experiments using 12-mm coverslips, 2.5×10^4^ HeLa cells were seeded one day before infection.

RACK1 RNAi cells were generated using the BLOCK-iT™ Inducible H1 Lentiviral RNAi System (Invitrogen) according to the manufacturer's instructions and then cultivated as described above. For the RACK1 RNAi two different target sequences were used: RACK1 RNAi 1 (start at 275): GGGTCACTCCCACTTTGTTAG and RACK1 RNAi 2 (start at 751): GGGATCTCAACGAAGGCAAAC.

Stably transduced HeLa cell lines expressing the RACK1 shRNA were selected using 100 µg/ml Zeocin^TM^ (Invitrogen). Individual clones were isolated and expanded, and cellular RACK1 levels were analyzed by Western blot.

### Antibodies

The following primary antibodies were used: mouse anti-human RACK1 (clone 20), mouse anti-human Erk (clone 16), mouse anti-human Ras (clone 18) (BD Transduction Laboratories); mouse anti-FLAG pre-coupled to beads (clone M2) (Sigma) rabbit anti-*Yersinia*, anti-YopK, anti-YopD, anti-YopB, anti-YopE, anti-Ail and anti-LcrV antisera (Agrisera, Sweden); mouse anti-human Tubulin (clone B-5-1-2) (Sigma). The following secondary antibodies were used: Alexa488 goat anti-mouse IgM, Alexa488 donkey anti-rabbit, and Alexa555 goat anti-rabbit (Molecular Probes, Invitrogen); HRP goat anti-mouse IgM (Invitrogen); HRP donkey anti-rabbit (GE Healthcare); HRP goat anti-mouse (DAKO).

### β-lactamase assay for determination of translocated YopK

One day before infection, HeLa cells were seeded on glass coverslips in a 24-well plate, and cytochalasin D (0.5 µg/ml) was added to the cells 30 min before infection to prevent bacterial internalization. Overnight cultures of *Y. pseudotuberculosis yopK* mutant [YPIII(pIB155)] and yopB mutant [YPIII(pIB604)] strains expressing YopK-βlac or YopK^AA^-βlac were diluted in fresh MEM and induced for Yop expression. HeLa cells were infected at a multiplicity of infection (MOI) of 20 for 3 hr. For microscopy, the FRET-based fluorescent β-lactamase substrate CCF2-AM (Invitrogen) was prepared according to the manufacturer's instructions and added directly to the cells. Cells were incubated for 1 hr in the dark at room temperature and then washed twice, fixed, and mounted using ultimate mounting medium (UMM) [46]. Samples were analyzed by fluorescence microscopy (Zeiss Axioskop), and images were captured using a CCD camera (Hamamatsu Orca C4742-95). The HeLa cells appeared green if there had been no translocation of Yop–β-lactamase fusions, whereas they were blue if they contained such fusions in their cytoplasm.

### Co-purification and co-precipitation

For YopD co-purification, 200 ml of *Y. pseudotuberculosis* YPIII(pIB29MEKByopD::His) encoding His-*yopD* and YPIII(pIB29MEKBD), both harbouring the *yopK* encoding pAH3 plasmid, were grown for 2.5 h in secretion permissive conditions in LB at 37°C. The cells were harvested and supernatants were filtered through a 45 µm SFCA filtering device (Nalgene). Additions were made to the cleared supernatants giving a final concentration of 20 mM Tris-HCl pH 7.4, 250 mM NaCl, 60 mM Imidazole, 10 mM MgCl_2_, and 5 mM CaCl_2_. The supernatants were passed through a 5 ml His-Trap FF column (GE Healthcare), washed with a buffer containing 20 mM Tris-HCl pH 7.4, 250 mM NaCl, 60 mM Imidazole 5 mM MgCl_2_, and 1 mM CaCl_2_, and eluted with a similar buffer containing 500 mM Imidazole. The final samples were analyzed using Western blot.

For YopK pulldown of proteins in bacterial supernatant, overnight cultures of *Y. pseudotuberculosis yopK* mutant [YPIII(pIB155)] and *yopK* mutant expressing YopK-FLAG [YPIII(pIB155, pAH11)] were diluted 1∶10 and grown in secretion permissive conditions in fresh BHI medium at 37°C. After centrifugation, the resulting culture supernatants were collected and incubated with beads coupled to anti-FLAG antibodies for 45 min at room temperature. The beads were subsequently collected by centrifugation and washed with RIPA buffer containing protease inhibitor (Roche). For detection of interaction of YopK–FLAG with proteins present in the bacterial supernatant, samples were analysed by SDS-PAGE and Western blot.

For YopK pulldown of proteins in Hela cell lysates a 10-µl portion of anti-FLAG beads was preincubated with 200 µl of culture supernatants from *Y. pseudotuberculosis* multiple yop mutant strain (YPIII(pIB29MEKBD) expressing or not expressing YopK-FLAG (grown as described above). The beads were thereafter washed and incubated with 120 µl of cleared HeLa cell lysate (3×10^6^ cells) for 45 min at room temperature. For reciprocal pull down experiments using purified recombinant RACK1, GST (pGEX-4T-1) and GST-RACK1 (pGEX-RACK1) were expressed in *E. coli* (BL21; Stratagene) and purified by standard methods recommended by the manufacturer (GE Healthcare). GST and GST-RACK1 on glutathione sepharose beads (GE healthcare) were washed with RIPA buffer containing protease inhibitor (Roche) and then incubated for 45 min at room temperature with a culture supernatant from *Y. pseudotuberculosis* multiple mutant expressing YopK-FLAG [YPIII(pIB29MEKBD, pAH11]. The different pull-down samples were centrifuged through a gradient (300 µl RIPA, 300 µl 17% glycerol in RIPA, 300 µl 27% sucrose/17% glycerol mixture in RIPA) for 1 min at 14 000 rpm. The pelleted material was boiled in sample buffer and separated by SDS-PAGE. Co-precipitation of RACK1 with YopK–FLAG and co-precipitation of YopK with GST-RACK1 was detected by Western blot.

### Animal infection and measurement of In Vivo bioluminescence

Eight-week-old female BALB/c mice (Scanbur, Denmark) were divided into three groups with five animals in each and then orally infected with *Y. pseudotuberculosis* wild-type (YPII/pCD1-Xen4), *yopK null* [YPIII(pCD155-Xen4)], or *yopK* ^AA^ [YPIII(pCD156-Xen4)]. The mice were deprived of food and water for 18 hr before oral infection. The *Yersinia* strains were grown overnight in LB at 26°C and subsequently resuspended in sterile tap water supplemented with 150 mM NaCl. The infection dose was determined by viable count and drinking volume measurements. The animals were inspected daily for signs of disease, and they were analyzed for light emission in an IVIS Spectrum (Caliper Life Sciences) on days 3, 8, 10, and 13 of infection. Prior to imaging, the mice were anesthetized with 2.5% isoflurane (IsoFlu®Vet, Orion Pharma Animal Health, Sweden) mixed with oxygen supplied from an XG1-8 gas system (Caliper Life Sciences). Images were acquired and analyzed using LivingImage software (Caliper Life Sciences). Total photon emission was recorded for each animal, and the light emitted from each individual mouse was quantified using the Region of Interest (ROI) tool.

### Determination of bacterial uptake

The *Y. pseudotuberculosis* strains were grown in LB over night at 26°C. For infection, bacteria were diluted in MEM and induced for Yop expression. HeLa cells (2.5×10^4^) were seeded on 12-mm glass coverslips the day before the experiment, and they were infected with a multiplicity of infection (MOI) of 20 for 30 min at 37°C in a humidified atmosphere containing 5% CO_2_. Intracellularly and extracellularly located bacteria were distinguished by using a previously described double-fluorescent labeling method [71], [72]. Samples were analyzed by fluorescence microscopy (Zeiss Axioskop). Numbers of extracellularly bound and total cell-associated bacteria were counted for 50 randomly selected cells per coverslip.

### Determination of effector translocation

Effector translocation was determined using a method based on the ability of *Pseudomonas aeruginosa* effector ExoS to modify eukaryotic Ras in infected cells [73]. For this, the *Y. pseudotuberculosis yopE* mutant strain expressing ExoS *in trans* [YPIII(pIB526), pTS103] was employed. A YopE defective background was chosen for this assay in order to avoid a repressing effect on pore formation and thereby influence of ExoS translocation efficiency [74]. Modification of Ras was visualized by Western blot. Protease protection and digitonin extraction of translocated *Yersinia* effector proteins were carried out as described elsewhere [49], [75]. Translocation of bacterial proteins was scored as the difference between YopE levels in digitonin-treated and untreated HeLa cells analyzed by Western blot.

### Cytotoxicity assay

HeLa cells were seeded on coverslips the day before infection. Overnight cultures of *Yersinia* strains were diluted in fresh MEM and induced for Yop expression. HeLa cells were infected with a multiplicity of infection (MOI) of 10–20 for 1 hr and were subsequently fixed with 2% PFA, washed in PBS, and the coverslips were mounted with Mowiol® (Calbiochem). Cytotoxicity (cell rounding) was assessed by microscopy as previously described [14].

### Immunofluorescence

HeLa cells were infected with *Yersinia* or subjected to a cell-spreading assay [47]. Thereafter cells were washed, fixed with 4% PFA, and permeabilized with 0.5% Triton X-100. To block non-specific binding, the specimens were overlaid with 0.1 M glycine followed by 5% serum from the secondary antibody host. The different samples were stained with indicated antibodies. The specimens were mounted in ultimate mounting medium (UMM) [46] supplemented with Citifluor (Ted Pella Inc.) or in Mowiol® (Calbiochem) and examined by fluorescence microscopy (Zeiss Axioskop) or confocal laser scanning microscopy (Leica instrument).

## Supporting Information

Figure S1 **Bacterial proteins in purified erythrocyte membranes after infection with** ***Y. pseudotuberculosis*** **.** Western blot control showing presence of YopD and YopB, but not LcrV or the bacterial outer membrane protein Ail, in the membrane fraction from erythrocytes infected with the *Y. pseudotuberculosis* wild-type strain.
(TIF)

Figure S2 **Mapping and characterization of the YopK–RACK1 interaction.** (A) Determination of interaction between different regions of the RACK1 protein and YopK_7-59_ and YopK^AA^_7-59_ respectively, using the yeast two-hybrid system as a read-out. pACTcDNA3 encodes the RACK1 fragment identified as a YopK-interacting protein in the initial yeast two hybrid screen. In the diagram, +++ indicates growth corresponding to that seen in strains with non-mutated YopK and RACK1, and – stands for no growth. (B) Determination of interaction between RACK1 and different regions of the YopK protein using the yeast two-hybrid system as a read-out. In the diagram, +++, ++, + and – correspond to degree of growth, where +++ indicate the most pronounced growth and – stands for no growth. (C) Determination of Yop translocation capacity of *Y. pseudotuberculosis yopK* mutants. HeLa cells were infected with the indicated strains and then treated with proteinase K. Thereafter, the cells were washed and lysed with digitonin or left untreated, whereafter the supernatants were subjected to SDS-PAGE and immunoblotted for YopE. The translocation deficient *yopD* mutant was included as negative control.
(TIF)

Figure S3 **Internalization of** ***Y. pseudotuberculosis*** **strains by RACK1 RNAi cell lines.** (A) Internalization of the indicated *Y. pseudotuberculosis* strains by HeLa control cells and two RACK1 RNAi cell lines obtained using oligos directed towards two different RACK1 target sequences. The number of internalized bacteria is presented as percent of the total number of cell-associated bacteria. The illustrated data represent the mean ± SEM of three independent experiments. (B) Internalization of the indicated *Y. pseudotuberculosis* strains by HeLa control cells and the indicated RACK1 RNAi cell lines. The number of internalized bacteria is presented as percent of the total number of cell-associated bacteria. The illustrated data represent the mean ± SEM of at least three independent experiments. P<0.05 *. *Inset* Downregulation of RACK1 expression in HeLa cells by RNAi. Lysates of HeLa control cells and different RACK1 RNAi clones were analyzed for RACK1 expression by Western blot using anti-RACK1 antibodies. The levels of RACK1 in the different RNAi cell lines are presented as expression relative to that observed in the HeLa control cells.
(TIF)

Figure S4 **Localization of RACK1 in cultured HeLa cells.** Localization of RACK1 in HeLa cells in culture was revealed by staining for RACK1 and analyzing by confocal microscopy.
(TIF)
