# Supplementary figures and images for: The RACK1 Signaling Scaffold Protein Selectively Interacts with Yersinia pseudotuberculosis Virulence Function
Source: PLoS One. 2011 Feb 10;6(2):e16784. doi: 10.1371/journal.pone.0016784 (PMC3037380; doi:10.1371/journal.pone.0016784)

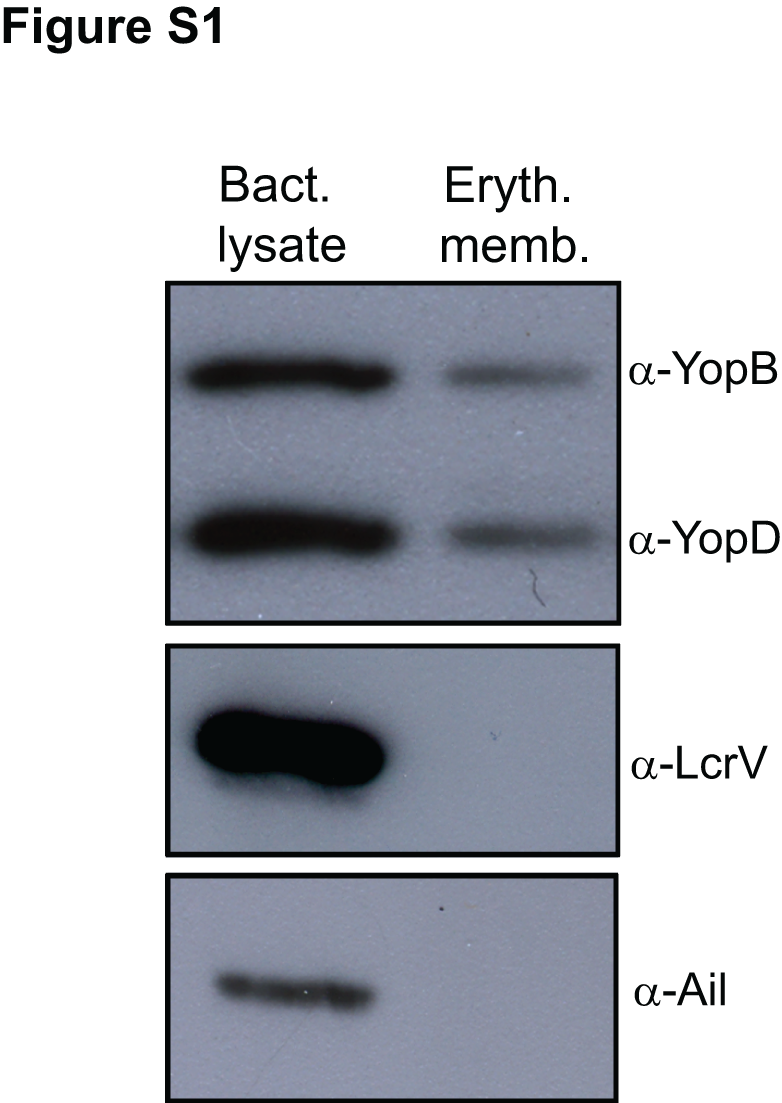

Supplement: Figure S1 — Bacterial proteins in purified erythrocyte membranes after infection with Y. pseudotuberculosis . Western blot control showing presence of YopD and YopB, but not LcrV or the bacterial outer membrane protein Ail, in the membrane fraction from erythrocytes infected with the Y. pseudotuberculosis wild-type strain. (TIF) [file pone.0016784.s001.tif]

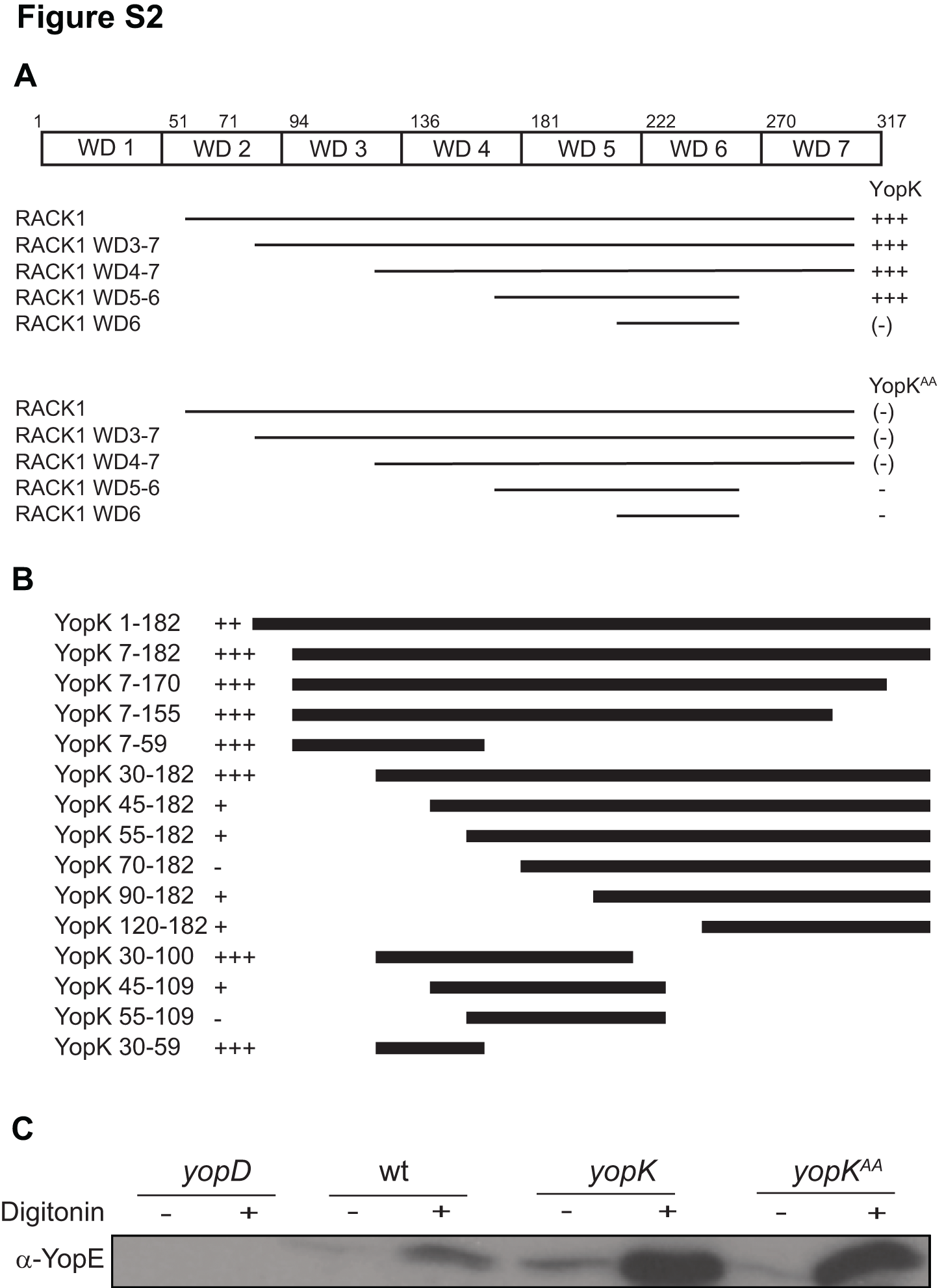

Supplement: Figure S2 — Mapping and characterization of the YopK–RACK1 interaction. (A) Determination of interaction between different regions of the RACK1 protein and YopK7-59 and YopKAA 7-59 respectively, using the yeast two-hybrid system as a read-out. pACTcDNA3 encodes the RACK1 fragment identified as a YopK-interacting protein in the initial yeast two hybrid screen. In the diagram, +++ indicates growth corresponding to that seen in strains with non-mutated YopK and RACK1, and – stands for no growth. (B) Determination of interaction between RACK1 and different regions of the YopK protein using the yeast two-hybrid system as a read-out. In the diagram, +++, ++, + and – correspond to degree of growth, where +++ indicate the most pronounced growth and – stands for no growth. (C) Determination of Yop translocation capacity of Y. pseudotuberculosis yopK mutants. HeLa cells were infected with the indicated strains and then treated with proteinase K. Thereafter, the cells were washed and lysed with digitonin or left untreated, whereafter the supernatants were subjected to SDS-PAGE and immunoblotted for YopE. The translocation deficient yopD mutant was included as negative control. (TIF) [file pone.0016784.s002.tif]

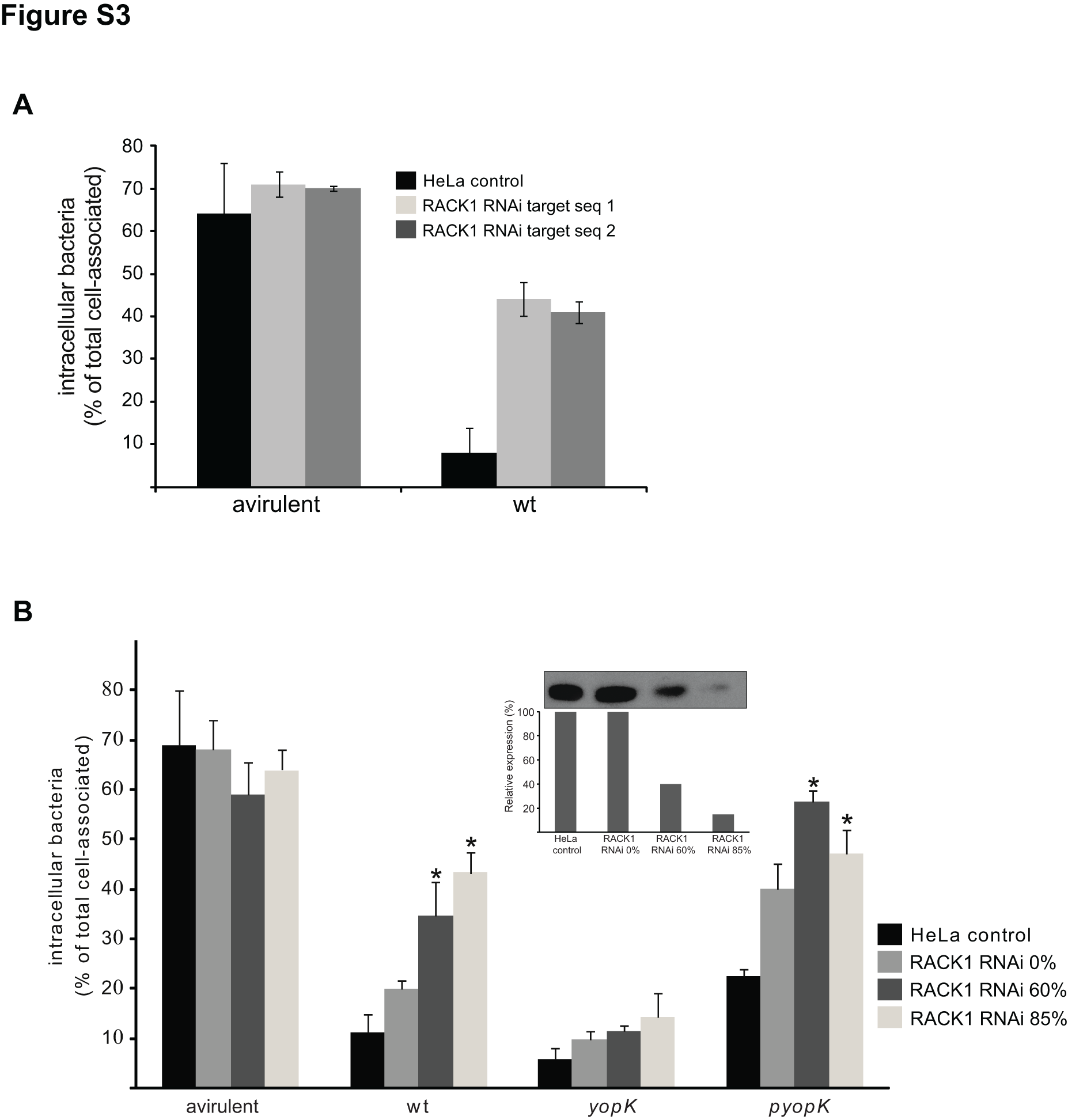

Supplement: Figure S3 — Internalization of Y. pseudotuberculosis strains by RACK1 RNAi cell lines. (A) Internalization of the indicated Y. pseudotuberculosis strains by HeLa control cells and two RACK1 RNAi cell lines obtained using oligos directed towards two different RACK1 target sequences. The number of internalized bacteria is presented as percent of the total number of cell-associated bacteria. The illustrated data represent the mean ± SEM of three independent experiments. (B) Internalization of the indicated Y. pseudotuberculosis strains by HeLa control cells and the indicated RACK1 RNAi cell lines. The number of internalized bacteria is presented as percent of the total number of cell-associated bacteria. The illustrated data represent the mean ± SEM of at least three independent experiments. P<0.05 *. Inset Downregulation of RACK1 expression in HeLa cells by RNAi. Lysates of HeLa control cells and different RACK1 RNAi clones were analyzed for RACK1 expression by Western blot using anti-RACK1 antibodies. The levels of RACK1 in the different RNAi cell lines are presented as expression relative to that observed in the HeLa control cells. (TIF) [file pone.0016784.s003.tif]

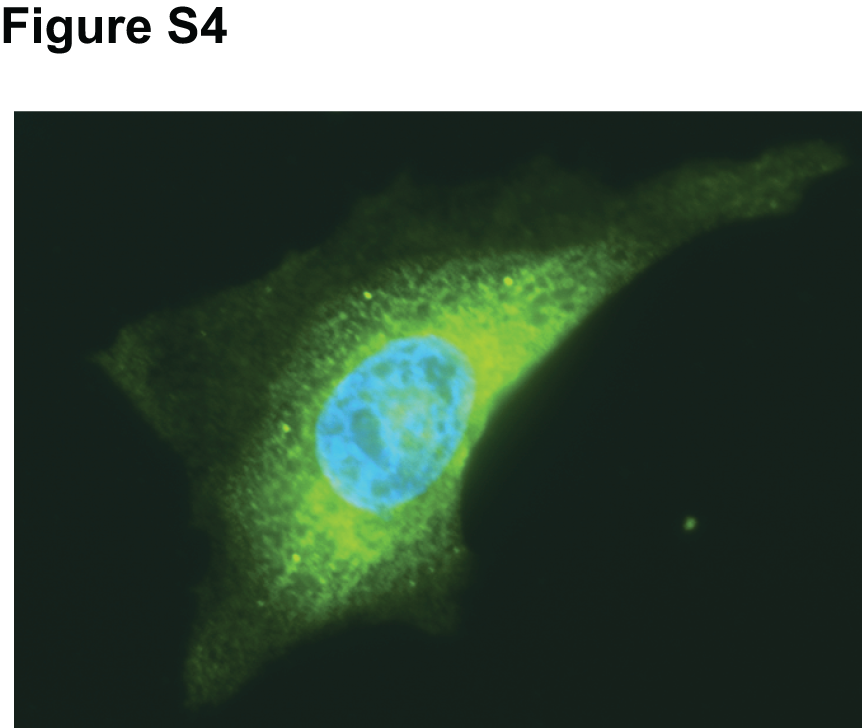

Supplement: Figure S4 — Localization of RACK1 in cultured HeLa cells. Localization of RACK1 in HeLa cells in culture was revealed by staining for RACK1 and analyzing by confocal microscopy. (TIF) [file pone.0016784.s004.tif]
